# Postoperative mortality among surgical patients with COVID-19: a systematic review and meta-analysis

**DOI:** 10.1186/s13037-020-00262-6

**Published:** 2020-10-12

**Authors:** Semagn Mekonnen Abate, Bahiru Mantefardo, Bivash Basu

**Affiliations:** 1 Department of Anesthesiology, College of Health Sciences and Medicine, Dilla University, PO.BOX:419, Dilla, Ethiopia; 2 Department of Internal Medicine, College of Health Sciences and Medicine, Dilla University, Dilla, Ethiopia; 3 Department of Anesthesiology, College of Health Sciences, University of Calcutta, Kolkata, India

**Keywords:** Perioperative, Mortality, Prevalence, Surgery, COVID-19

## Abstract

**Background:**

The coronavirus disease 2019 (COVID-19) pandemic puts perioperative providers and staff at risk of viral exposure to severe acute respiratory syndrome coronavirus-2 (SARS-CoV-2) during aerosol-generating procedures, particularly in asymptomatic carriers.However, the perioperative risk for adverse outcomes in SARS-CoV-2 infected patients remain uncertain and the topic of debate. The current study was designed to determine the postoperative mortality in COVID-19 patients based on a systematic review and meta-analysis of the global published peer-reviewed literature.

**Methods:**

A comprehensive search was conducted in PubMed/Medline; Science direct and LILACS from December 29, 2019, to August15, 2020, without language restriction. All observational studies reporting the prevalence of mortality were included while case reports and reviews were excluded. The data from each study were extracted with two independent authors with a customized format excel sheet and the disagreements were resolved by the third author. The methodological quality of included studies was evaluated using a standardized critical appraisal Tool adapted from the Joanna Briggs Institute.

**Results:**

A total of 715 articles were identified from different databases and 45 articles were selected for evaluation after the successive screening. Twenty-three articles with 2947 participants were included. The meta-analysis revealed a very high global rate of postoperative mortality among COVID-19 patients of 20% (95% CI: 15 to 26) and a postoperative ICU admission rate of 15% (95% confidence interval (CI):10 to 21).

**Conclusion:**

The unexpected high postoperative mortality rate in SARS-CoV-2 infected patients of 20% in the global literature mandates further scrutiny in assuring appropriate surgical indications and perioperative surgical safety measures in this vulnerable cohort of patients.

**Registration:**

This systematic review and meta-analysis was registered in Prospero’s international prospective register of systematic reviews (CRD42020203362) on August 10, 2020.

## Background

The severe acute respiratory syndrome virus-2 (SARS-CoV-2) virus that causes coronavirus disease 2019 (COVID-19) was identified in Wuhan, Hubei province of China in December 2019 by the Chinese Center for Disease and Prevention from the throat swab of a patient [1]. The Coronavirus infection mainly affects the respiratory system and is presented with fever, dry cough, and difficulty of breathing, and lately, the patient may deteriorate associated with pneumonia and acute respiratory distress syndrome [1–4] despite recent evidence on the clinical manifestation of the gastrointestinal tract, cardiac, dermatologic, cardiac and central nervous system [5–12].

World Health Organization (WHO) situational report revealed that there were more than 20 million laboratory-confirmed cases and 700 thousand deaths globally as of August13, 2020 [13]. The American region accounted for the highest number of cases and deaths which was 10 million and 400 thousand respectively [13]. The European region accounted for the second-highest confirmed cases and death which were more than 3 million confirmed cases and 200 thousand deaths. Though the COVID-19 pandemic has emerged in the Western Pacific region, China, Hwan city, the number of infected cases, and deaths was the lowest as compared to the American and European regions [13]. The number of laboratory-confirmed cases and deaths in the African region was the lowest for the last couple of months but the rate of spreading in this region is increasing at an alarming rate and expected to be very high in the next couple of months if it continues as this rate [13–15].

The last couple of months’ reports in Ethiopia were very low but there were many cases in short periods which is approximately 1000 cases and 10 deaths per day. It is estimated that the number even may be very high because the diagnosis is limited only in big cities. The huge discrepancies among counties on number of infected case and mortality may be related with testing capacity and some countries fail to report the actual data consistently due to different reasons.

The challenge of COVID-19 is very high globally due to a lack of proven treatment and the complexity of its transmission [16–20]. However, the impact is more catastrophic for low and middle-income countries because of very poor health care system, high illiteracy and low awareness of the disease and its prevention, lack of skilled health personnel, scarce Intensive Care Unit, a limited number of mechanical ventilators, and prevalence of co-morbidities/infection along with malnutrition [15, 20–22].

Epidemiological studies showed that patients with co-morbidities including (Asthma, COPD, Tuberculosis, Pneumonia, Acute respiratory distress syndrome (ARDS), Diabetes mellitus, hypertension, renal disease, hepatic disease, and cardiac disease), history of smoking, and history of substance use, male gender and age greater than 60 years were more likely to die or develop undesirable outcomes [23–26].

The outcomes of patients with coronavirus infection undergoing surgery are very variable. Studies revealed that in-hospital mortality of patients with COVID-19 was very high which varied from 1 52% of the hospitalized patient [27, 28].

Body of evidence showed that patients visiting the health institution during the COVID-19 pandemic decrease significantly despite requiring medical care which affects significantly the non-COVID-19 patients’ hospital admission [29–33].

The COVID-19 pandemic imposes a significant challenge on health care delivery along with economic, social, and mental health crisis [15–18, 30, 33–42].

Surgery during the COVID-19 outbreak is challenging to the patient, health care workers, and non-COVID-19 patients [34, 35, 38, 43–46] particularly for low and middle-income countries where the limping health care systems were broken with low testing capacity, sub-optimal postoperative care, lack of anesthesia machine filters and limited personal protective equipment [47–50].

Evidence revealed that mortality of patients hospitalized with COVID-19 was very high which is strongly associated with the presence of comorbidities, smoking, and substance use [23, 25, 34, 39, 45, 51, 52].

Some studies showed that perioperative mortality of patients with COVID-19 was very high [30, 32, 45, 53–55] while some studies failed to identify significant mortality among patients with COVID-19 undergoing surgical procedures [28, 52, 56, 57].

Investigating the global prevalence and determinants of perioperative outcomes among patients with COVID-19 undergoing a surgical procedure is very important to reduce patient mortality and morbidity through varies strategies including but not limited to the provision of alternative non-surgical intervention for a moderate and severe case, increasing the number of ICU beds, mechanical ventilator, skilled professionals, and integrated monitors and reducing possible risk factors. Therefore, this systematic review and meta-analysis aimed to provide global evidence on the prevalence and determinants of perioperative outcomes among patients with COVID-19 undergoing surgical procedures.

## Methods

### Protocol and registration

The systematic review and meta-analysis were conducted based on the Preferred Reporting Items for Systematic and meta-analysis (PRISMA) protocols [58]. This systematic review and meta-analysis were registered in Prospero’s international prospective register of systematic reviews (CRD42020203362) on August 10, 2020.

### Eligibility criteria

All observational (case series, cross-sectional, cohort, and case-control) studies reporting the prevalence of mortality and its determinants among surgical patients with coronavirus disease (COVID-19) were included while studies that didn’t report the prevalence of mortality among surgical patients with COVID-19, articles that didn’t report full information for data extraction, articles with different outcomes of interest, studies with a methodological score less than 50 %, studies with randomized controlled trials, and Systemic review study design were excluded. The primary outcomes of interest were the global prevalence of postoperative mortality and the rate of postoperative ICU admission among patients with COVID-19 worldwide. The prevalence of comorbidities, prevalence of postoperative complications and lengths of hospital stay were secondary outcomes.

### Search strategy

The search strategy was conducted to explore all available published and unpublished studies among surgical COVID-19 patients admitted to the hospital from December 2019 to August 2020 without language restrictions. A comprehensive search was employed in this review. An initial search on PubMed/Medline, Science Direct and Cochrane Library was carried out followed by an analysis of the text words contained in Title/Abstract and indexed terms. A second search was undertaken by combining free text words and indexed terms with Boolean operators. The third search was conducted with the reference lists of all identified reports and articles for additional studies. Finally, an additional and grey literature search was conducted on Google scholars. The databases were searched with the following search terms using PICO strategy by combining with AND, OR Boolean operators as COVID-19 OR coronavirus OR SARS-CoV-2 AND surgery OR operation OR preoperative OR perioperative OR postoperative AND outcomes OR mortality OR death OR morbidity OR hospital stay OR complication OR infection OR ARDS AND anesthesia OR general OR regional OR spinal.

**Fig. 1:**
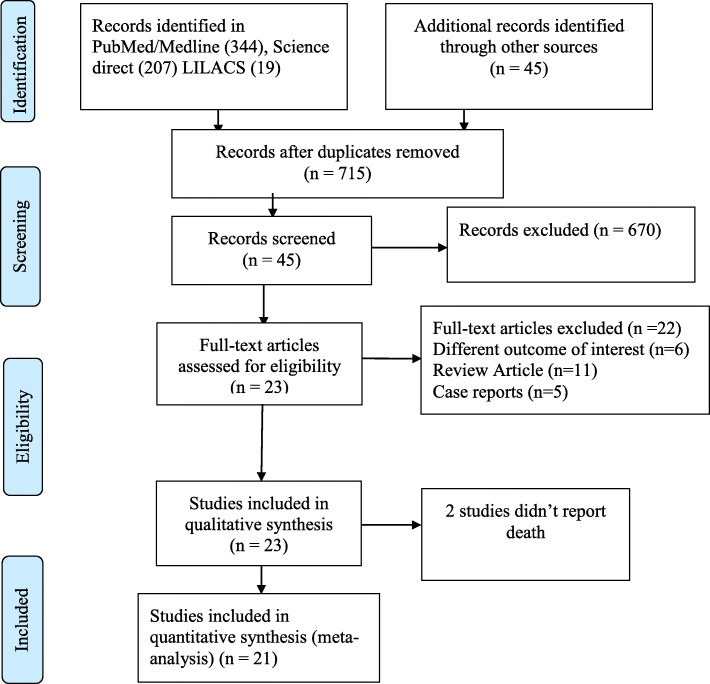
Prisma flow chart

The final search results were shown with the Prisma flow diagram **(**Fig. 1**).**

### Data extraction

The data from each study were extracted with two independent authors with a customized format excel sheet. The disagreements between the two independent authors were resolved by the third author. The extracted data included: Author names, country, date of publication, sample size, mortality, postoperative mechanical ventilator, the number of days on a mechanical ventilator, presence of co-morbidities, and complication. Finally, the data were then imported for analysis in R software version 4.0.2 and STATA 16.

### Assessment of methodological quality

Articles identified for retrieval were assessed by two independent Authors for methodological quality before inclusion in the review using a standardized critical appraisal Tool adapted from the Joanna Briggs Institute (Supplemental Table 1). The disagreements between the Authors appraising the articles were resolved through discussion. Articles with average scores greater than 50 % were included for data extraction.

### Data analysis

Data analysis was carried out in R statistical software version 4.0.2 and STATA 16. The pooled global prevalence of mortality, comorbidity, and complication among surgical patients with COVID-19 was determined with a random effect model as there was substantial heterogeneity. The Heterogeneity among the included studies was checked with forest plot, χ2 test, I^2^ test, and the *p*-values. Substantial heterogeneity among the included studies was investigated with subgroup analysis.

Publication bias was checked with a funnel plot and the objective diagnostic test was conducted with Egger’s correlation, Begg’s regression tests, and Trim and fill method. Furthermore, moderator analysis was carried out to identify the independent predictors of mortality among corona cases.

## Results

### Selection of studies

A total of 715 articles were identified from different databases and 45 articles were selected for evaluation after the successive screening. Twenty-three articles with 2947 participants were included and the rest were excluded with reasons [34, 37–40, 56, 59–74] (Fig. 1).

### Description of included studies

Twenty-three Articles with 2947 participants were included in the review while twenty-one studies were included in the meta-analysis for the prevalence of mortality. Studies with the prevalence of mortality and/or prevalence of comorbidity and prevalence of complications among surgical patients with COVID-19 were included and the characteristics of each included studies were described in **(**Table 1) and the rest were excluded with reasons.

**Table 1:** Description of included studies

Author	Study period	Country	sample	Category	Urgency	quality	P(95% CI
Bhangu et al.	Jan 1 to March 31, 2020	UK	1128	Any	Any	Low risk	24 [21,26]
Cai et al. [53]	February, 2020	China	7	Any	Any	Low risk	43 [10,82]
Casanova et al. [28]	March 11 to May 15, 2020	Portugal	148	Cardiac	Emergency	Low risk	1 [0,5]
Cheung et al. [75]	March 1 to May 22, 2020	USA	10	Orthopedics	Emergency	Low risk	10 [0,45]
Doglietto et al. [76]	Feb 1, April 23	Italy	41	Any	Any	Low risk	20 [9,35]
Dursun et al. [30]	March 10 to May 20, 2020	Turkey	200	Gynecology	Elective	Low risk	12 [8,17]
Egol et al. [36]	Feb 1 to April 15, 2020	USA	253	Orthopedics	Elective	Low risk	7 [4.11]
Kayani et al. [44]	Feb 1 to April, 2020	UK	82	Orthopedics	Elective	Low risk	30 [21,42]
LeBrun et al. [54]	March 20 to April 24, 2020	USA	9	Orthopedics	Any	Low risk	78 [40,97]
Lei et al. [77]	Jan 1 to Feb 5, 2020	China	34	Any	Any	Low risk	21 [9,38]
Li et al. [78]	Jan 1 to Feb 5, 2020	China	54	Any	Emergency	Low risk	15 [7,27]
Macey et al. [65]	Dec to March 2020	UK	76	Orthopedics	Any	Low risk	28 [18,39]
Martino et al. [79]	Feb 17 to March 31, 2020	Spain	15	Any	Any	Low risk	20 [4,48]
Mi et al. [80]	Jan 1 to Feb 27,2020	China	3	Orthopedics	Any	Low risk	20 [4,48]
Pai et al. [32]	March 24 to May 31, 2020	India	184	Any	Elective	Low risk	20 [14,26]
Peng et al. [55]	January 2020	China	11	Thoracic	Any	Low risk	27 [6,61]
Rajasekaran et al. [27]	March 12 to May 12, 2020	UK	56	Orthopedics	Any	Low risk	4 [0,12]
Santiago et al. [81]	March to May, 2020	Spain	126	Gynecology	Elective	Low risk	12 [7,19]
Seeliger et al. [27]	March 1 to May 23, 2020	France	13	Any	Emergency	Low risk	92 [64,100]
Sobti et al. [51]	March 1 to May 31, 2020	UK	206	Orthopedics	Any	Low risk	4 [2,8]
Stevenson et al. [82]	March 4 to May 22, 2020	UK	100	Orthopedics	Elective	Low risk	7 [3,14]
Stoneham et al. [57]	March 1 to June 12,020	UK	48	Orthopedics	Elective	Low risk	–
Zhang et al. [52]	Jan 1 to March 20, 2020	China	133	Obstetrics	Both	Low risk	–

The included studies were published from December 16, 2019, to June 1, 2020, with sample sizes, ranged from 3 to 1128. The mean (±SD) ages of the included studies varied from 33.7 ± 2.75 to 85 ± 8.75 years.

The majority of the included studies were conducted United Kingdom (7), China (6), USA (3), and Spain (2) [23, 26, 83–102]. Five studies were conducted in India, Italy, France, Portugal, and Turkey. Twenty-one of the included studies reported the prevalence of mortality among surgical patients with COVID-19 while two of the included studies didn’t report the prevalence of mortality among surgical COVID-19 patients in the hospital. The prevalence of mortality in surgical patients with COVID-19 from the included studies varied from 1 to 92%.

Ten studies with 2134 participants reported the prevalence of comorbidity including hypertension, diabetes mellitus, cardiovascular disease, and dementia as the major comorbidity among surgical patients with COVID-19 while ten studies with 1920 participants reporting the prevalence of complications including pulmonary, acute kidney injury, myocardial Infarction, Thromboembolic disease, infection, and deep wound infection as the major complications.

The prevalence of ICU admission was reported in ten of the included studies while the overall length of hospital stay was reported in thirteen of the included studies.

### Meta-analysis

#### Global prevalence of perioperative mortality

**Fig. 2:**
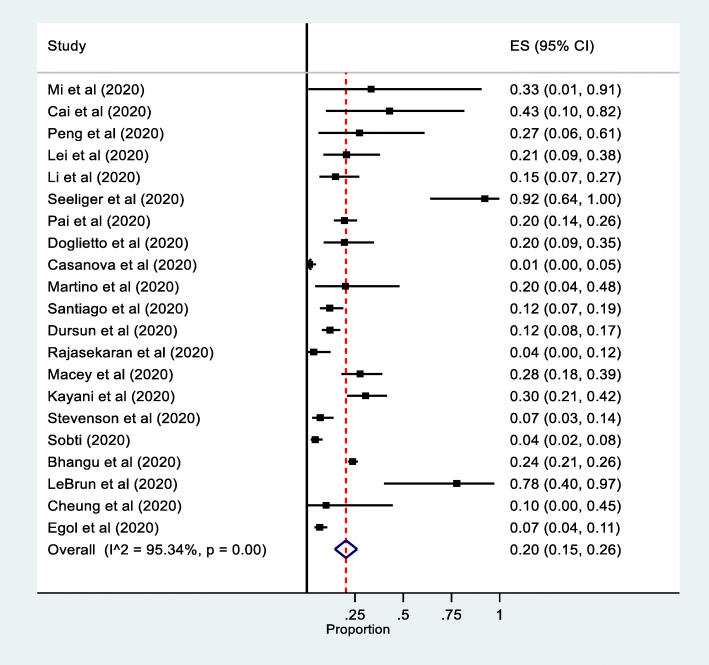
Forest plot for the prevalence of mortality among hospitalized patients with COVID: The mid-point of each line illustrates the prevalence; the horizontal line indicates the confidence interval, and the diamond shows the pooled prevalence

Twenty-one studies reported the prevalence of perioperative mortality among surgical patients with COVID-19. The pooled prevalence of perioperative mortality was 20% (95% CI: 15 to 26, 21 studies, and 2756 participants) **(**Fig. 2**).**

**Fig. 3:**
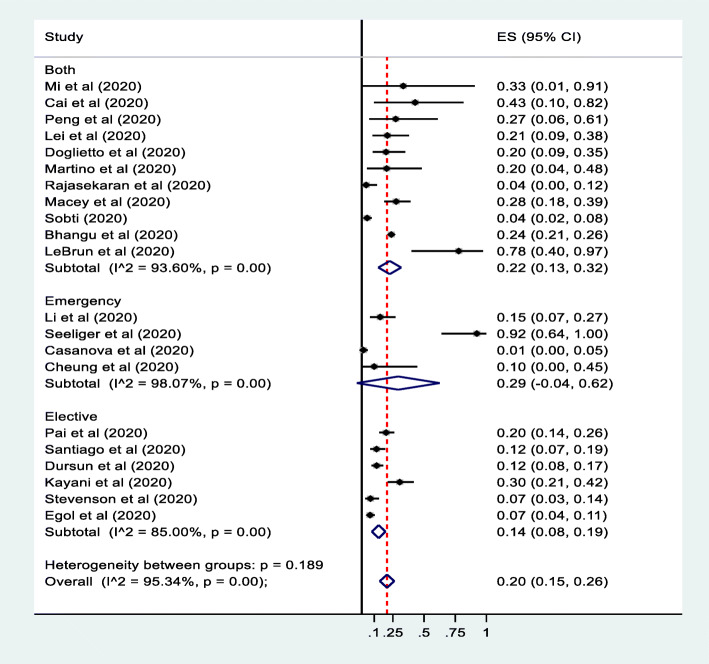
Forest plot for the subgroup analysis of the prevalence of perioperative mortality among surgical patients with COVID-19 by the urgency of surgery: The midpoint of each line illustrates the prevalence; the horizontal line indicates the confidence interval, and the diamond shows the pooled prevalence. Forest plot for the prevalence of perioperative morbidity among surgical patients with COVID-19: The midpoint of each line illustrates the prevalence; the horizontal line indicates the confidence interval, and the diamond shows the pooled prevalence

The sub-group analysis was conducted by country, surgical category, and urgency of surgery. The sub-group analysis revealed that perioperative mortality was the highest among emergency surgical patients, 29% (95% confidence interval (CI):-4 to 62%) **(**Fig. 3**).** The perioperative mortality among surgical patients with COVID-19 was found to be higher in France followed by the USA, 92% (95% confidence interval (CI): 64 to 100) and 29% (95% confidence interval (CI):-4 to 62) respectively (Supplemental Figure 1). Besides, the perioperative mortality was the highest among any surgical category followed by Orthopedics (Supplemental Figure 2).

### Prevalence of perioperative morbidity

The meta-analysis revealed that the prevalence of perioperative morbidity among surgical patients with COVID-19 was 18% (95% CI: 10 to 25, 10 studies, 2134 participants) (Fig. 3). The sub-group analysis revealed that dementia, DM, and hypertension were the most common comorbidities among surgical patients with COVID-19, 78% (95% confidence interval (CI):40 to 97), 20% (95% confidence interval (CI): 9 to 35) and 15% (95% confidence interval (CI):4 to 25) respectively (Supplemental Figure 3).

### Prevalence of perioperative complication

**Fig. 4:**
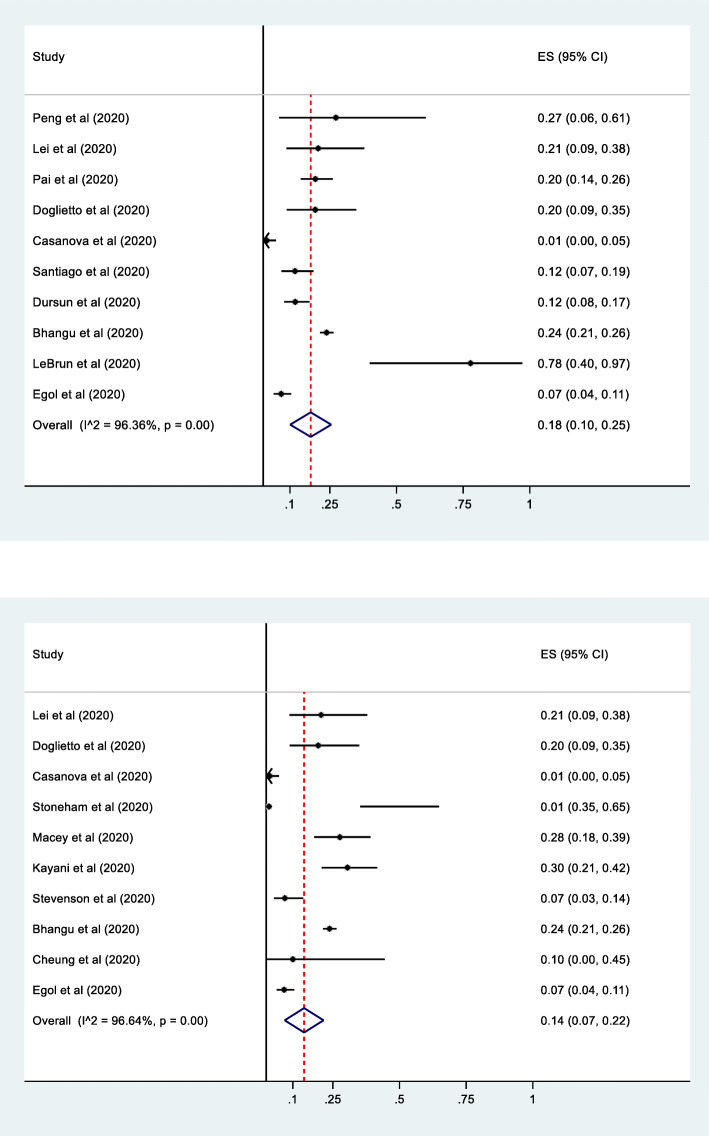
Forest plot for the prevalence of perioperative complication among surgical patients with COVID-19: The midpoint of each line illustrates the prevalence; the horizontal line indicates the confidence interval, and the diamond shows the pooled prevalence

The pooled prevalence of perioperative complications was estimated by taking the commonest reported complication among others. The meta-analysis showed that the pooled prevalence of perioperative complications among surgical patients with COVID-19 was 14% (95% confidence interval (CI):7 to 22, ten studies, and 1920) participants **(**Fig. 4**).** The subgroup analysis revealed that Thromboembolic complication, infection, and pulmonary complications were the most common perioperative complications among surgical patients with COVID-19 (Supplemental Figure 4).

### Mean duration of hospitalization

**Fig. 5:**
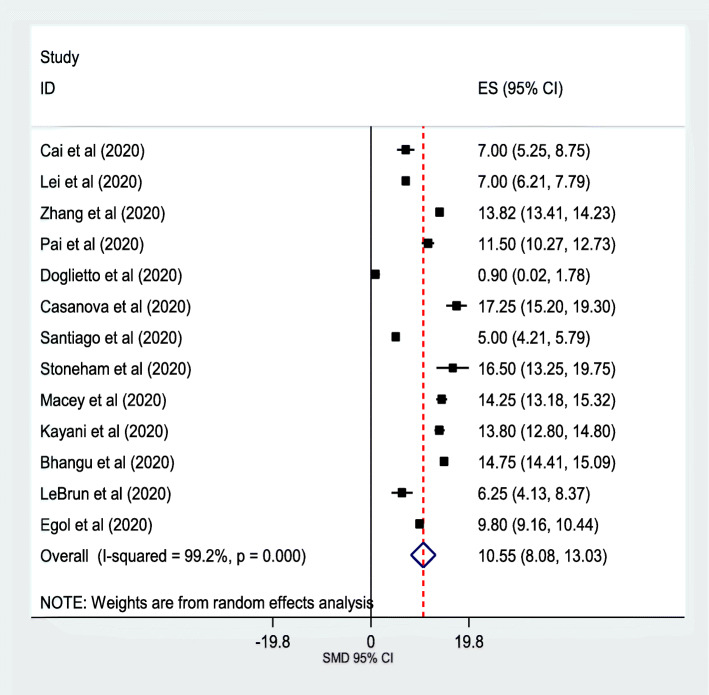
Forest plot for the length of hospital stay among surgical patients with COVID-19: The midpoint of each line illustrates the prevalence; the horizontal line indicates the confidence interval, and the diamond shows the pooled prevalence

The pooled mean duration of hospitalization was estimated from included studies mean duration of hospitalization. The meta-analysis revealed that the mean duration of Hospitalization was 10.55 (95% confidence interval (CI): 8.08 to 13.03, 13 studies, 2269 participants) (Fig. 5).

### Rate of postoperative ICU admission

**Fig. 6:**
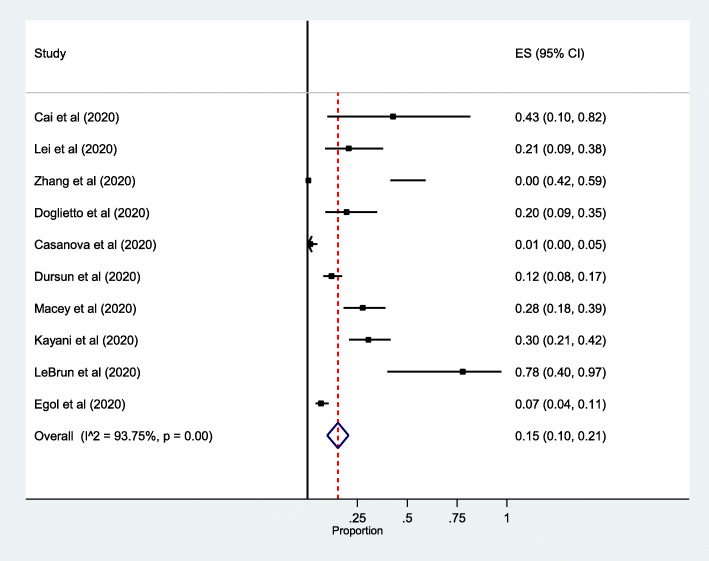
Forest plot for rates postoperative ICU admission among surgical patients with COVID-19: The midpoint of each line illustrates the prevalence; the horizontal line indicates the confidence interval, and the diamond shows the pooled prevalence

The meta-analysis revealed that the rate postoperative ICU admission among surgical patients with COVID-19 was 15% (95% confidence interval (CI):10 to 21, 10 studies, 983 participants) (Fig. 6).

#### Prevalence of clinical presentation

**Fig. 7:**
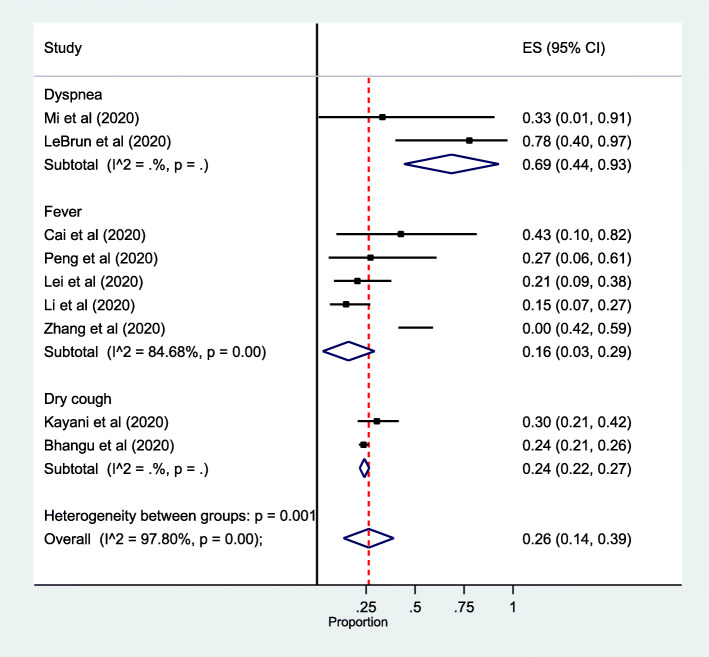
Forest plot for subgroup analysis of the prevalence of clinical presentation among surgical patients with COVID-19 by types of presentations: The midpoint of each line illustrates the prevalence; the horizontal line indicates the confidence interval, and the diamond shows the pooled prevalence

Plenty of clinical manifestations were mentioned in included studies including fever, dry cough, dyspnea, sore throat, and diarrhea. The prevalence of clinical presentation among surgical patients with COVID-19 was 26% (95% confidence interval (CI): 14 to 39, 9 studies, and 1461 participants) (Fig. 7).

### Meta-regression

The meta-analysis showed a substantial heterogeneity between the included studies which entails meta-regression to identify the sources of heterogeneity. Regression Analysis was run for perioperative outcomes with mean age, length of hospital stay, and urgency of surgery moderators. However, none of the moderators showed significant association (*P*-Value > 0.05).

### Sensitivity analysis and publication bias

**Fig. 8:**
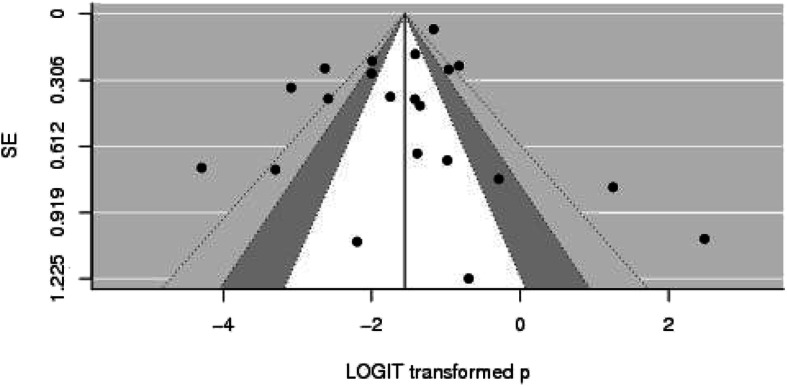
Funnel plot to assess publication bias. The vertical line indicates the effect size whereas the diagonal line indicates the precision of individual studies with a 95% confidence interval

Sensitivity analysis was conducted to identify the most influential study on the pooled summary effect and we didn’t find a significant influencing summary effect. The funnel plot didn’t show significant publication bias. Besides, egger’s regression and Begg’s correlation rank correlation failed to show a significant difference (*p* = 0.339 and *p* = 2.862) respectively (Fig. 8).

## Discussion

The meta-analysis revealed that the global prevalence of perioperative mortality among surgical patients with COVID-19 was 20% (95% CI: 15 to 26). The sub-group analysis showed that perioperative mortality among COVID-19 patients was very high in patients with emergency surgery, and among studies that included different surgical categories. However, the prevalence of perioperative mortality among COVID-19 patients was the highest in patients with orthopedics surgical procedures. This discrepancy could be explained by the inclusion of low powered included studies in the case of any surgical procedures.

The lower prevalence of perioperative mortality among orthopedic patients compared to other surgical procedures could be because these patients underwent the surgical procedure under spinal anesthesia which decreases airway manipulation, aspiration, postoperative delirium, thromboembolic disease and also improves rapid oral intake and early ambulation.

The meta-analysis showed that the prevalence of comorbidity among surgical patients was with COVID-19 was 18% (95% CI: 10 to 25) which is in line with the results of included studies [28, 30, 36, 54, 76, 77, 81]. The subgroup analysis showed that dementia was the most prevalent comorbidity followed by hypertension and Diabetes Mellitus. However, the finding of this study was contrary to other studies conducted on the prevalence of comorbidities among patients with COVID-19 where hypertension was the most prevalent comorbidity among patients with COVID-19 [28, 32, 77]. This discrepancy might be explained by the inclusion of only one study in this meta-analysis that reported a high prevalence of dementia compared to other comorbidities.

Many complications were mentioned in the literature in patients with COVID-19 who underwent surgical procedures [28, 36, 44, 45, 57, 75–77, 82]. The meta-analysis revealed that thromboembolic disease, pulmonary complications, infection, and deep wound infection were the commonest perioperative complications. All these complications were more likely associated with low immunity and prolonged immobility while patients were on a mechanical ventilator.

The meta-analysis showed that the pooled mean duration of hospitalization was 10.55 (95% confidence interval (CI): 8.08 to 13.03) which is comparable with the findings of the included studies [28, 36, 44, 45, 57, 75–77, 82].

### Quality of evidence

The methodological quality of included studies was moderate to high quality as depicted with Joanna Briggs Institute assessment tool for meta-analysis of observational studies. However, substantial heterogeneity associated with dissimilarities of included studies in sample size, design, and location could affect the allover quality of evidence.

### Limitation of the study

The meta-analysis included studies with moderate to high methodological quality. However, some of the included studies were low powered and the majority of studies included in this review didn’t report data on comorbidity and risk factors to investigate the independent predictors. Besides, there were a limited number of studies in some countries and it would be difficult to provide conclusive evidence with results pooled from fewer studies.

### Implication for practice

Body of evidence revealed that perioperative mortality; morbidity and complications were very high among patients with COVID-19. This is a huge challenge especially in resource-limited settings where there are a limited number of ICU beds, mechanical ventilator, integrated patient monitor, skilled professionals combined with malnutrition, and communicable disease. Therefore, a mitigating strategy is required by different stakeholders to combat the catastrophic impacts of the COVID-19 pandemic through creating awareness about preventive measures, implementing protocols for supportive management, management of comorbidities, and prevention of complications.

### The implication for further research

The meta-analysis revealed that perioperative mortality, complication, rate of ICU admission among surgical COVD-19 patients was very high. However, the included studies were too heterogeneous, low powered, and cross-sectional studies also don’t show a temporal relationship between mortality and its determinants. Therefore, further observational and randomized controlled trials are required.

## Conclusion

The meta-analysis revealed that the prevalence of mortality, perioperative complication, and rate of intensive care unit admission was very high. The meta-analysis showed that there is one death for every five COVID-19 patients undergoing surgical procedures which entail mitigating strategies to decrease perioperative mortality, infection transmission to health care workers, and non-COVID-19 patients; provide less risky anesthetic techniques and alternative management other than surgical procedures. Besides, there have to be guidelines to operate or not to operate high patients with COVID-19 for elective and urgent surgeries.

## Supplementary information

**Additional file 1: Supplemental Table 1.** methodological quality of included studies

**Additional file 2: Supplemental Figure 1.** Forest plot for the global prevalence of perioperative mortality among surgical patients with COVID-19 by types of surgery: The midpoint of each line illustrates the prevalence; the horizontal line indicates the confidence interval, and the diamond shows the pooled prevalence.

**Additional file 3: Supplemental Figure 2.** Forest plot for the global prevalence of perioperative mortality among surgical patients with COVID-19 by urgency of surgery: The midpoint of each line illustrates the prevalence; the horizontal line indicates the confidence interval, and the diamond shows the pooled prevalence.

**Additional file 4: Supplemental Figure 3.** Forest plot for the subgroup analysis of prevalence of perioperative comorbidity among surgical patients with COVID-19 by types of comorbidity: The midpoint of each line illustrates the prevalence; the horizontal line indicates the confidence interval, and the diamond shows the pooled prevalence.

**Additional file 5: Supplemental Figure 4.** Forest plot for sub-group analysis of prevalence of perioperative complication among surgical patients with COVID-19 by types of complication: The midpoint of each line illustrates the prevalence; the horizontal line indicates the confidence interval, and the diamond shows the pooled prevalence

## Data Availability

Data and material can be available where appropriate.
