# Supplementary material for: Postoperative mortality among surgical patients with COVID-19: a systematic review and meta-analysis
Source: Patient Saf Surg. 2020 Oct 12;14:37. doi: 10.1186/s13037-020-00262-6 (PMC7549731; doi:10.1186/s13037-020-00262-6)
Supplement: Supplementary file 1 — Additional file 1: Supplemental Table 1. methodological quality of included studies [file 13037_2020_262_MOESM1_ESM.docx]

## Supplemental Table 1: methodological quality of included studies

| Author(s) | Q1 | Q2 | Q3 | Q4 | Q5 | Q6 | Q7 | Q8 | Score |
| --- | --- | --- | --- | --- | --- | --- | --- | --- | --- |
| Mi et al | Y | Y | Y | Y | Y | N | Y | N | 6 |
| Cai et al | Y | Y | Y | N | N | N | Y | Y | 5 |
| Peng et al | Y | Y | Y | Y | Y | N | Y | N | 6 |
| Lei et al | Y | Y | Y | N | N | N | Y | Y | 5 |
| Li et al | Y | Y | Y | N | N | N | Y | Y | 5 |
| Zhang et al | Y | Y | Y | Y | N | N | N | N | 4 |
| Seeliger et al | Y | Y | Y | Y | Y | N | Y | N | 6 |
| Pai et al | Y | Y | Y | N | N | N | Y | Y | 5 |
| Doglietto et al | Y | Y | Y | Y | N | N | Y | N | 5 |
| Casanova et al | Y | Y | Y | Y | N | N | Y | N | 5 |
| Martino et al | Y | Y | Y | Y | N | Y | N | Y | 6 |
| Santiago et al | Y | Y | Y | Y | N | N | Y | N | 5 |
| Dursun et al | Y | Y | Y | N | N | Y | Y | Y | 6 |
| Stoneham et al | Y | Y | Y | N | N | N | Y | Y | 5 |
| Rajasekaran et al | Y | Y | Y | Y | N | N | Y | N | 6 |
| Macey et al | Y | Y | Y | N | N | N | Y | Y | 5 |
| Kayani et al | Y | Y | Y | N | N | N | Y | Y | 5 |
| Stevenson et al | Y | Y | Y | N | N | N | Y | Y | 5 |
| Sobti | Y | Y | Y | N | N | N | Y | Y | 5 |
| Bhangu et al | Y | Y | Y | Y | Y | Y | Y | Y | 8 |
| LeBrun et al | Y | Y | Y |  | N | N | Y | N | 6 |
| Cheung et al | Y | Y | Y | N | N | N | Y | Y | 5 |
| Egol et al | Y | Y | Y | Y | N | N | Y | Y | 6 |

Q: question; Y: yes; N: No, NA: not applicable
