# Supplementary material for: Postoperative mortality among surgical patients with COVID-19: a systematic review and meta-analysis
Source: Patient Saf Surg. 2020 Oct 12;14:37. doi: 10.1186/s13037-020-00262-6 (PMC7549731; doi:10.1186/s13037-020-00262-6)

**Fig 2:** Forest plot for the global prevalence of perioperative mortality among surgical patients with COVID-19: The midpoint of each line illustrates the prevalence; the horizontal line indicates the confidence interval, and the diamond shows the pooled prevalence.

**Supplemental Fig 1:** Forest plot for subgroup analysis of the prevalence of mortality among surgical patients with COVID-19 by country. The midpoint of each line illustrates the prevalence; the horizontal line indicates the confidence interval, and the diamond shows the pooled prevalence

**Fig 3:** Forest plot for the global prevalence of perioperative mortality among surgical patients with COVID-19 by types of surgery: The midpoint of each line illustrates the prevalence; the horizontal line indicates the confidence interval, and the diamond shows the pooled prevalence.

**Fig 4:** Forest plot for the global prevalence of perioperative mortality among surgical patients with COVID-19 by urgency of surgery: The midpoint of each line illustrates the prevalence; the horizontal line indicates the confidence interval, and the diamond shows the pooled prevalence.

**Fig 4:** Forest plot for the prevalence of perioperative comorbidity among surgical patients with COVID-19: The midpoint of each line illustrates the prevalence; the horizontal line indicates the confidence interval, and the diamond shows the pooled prevalence.

**Fig 5:** Forest plot for the subgroup analysis of prevalence of perioperative comorbidity among surgical patients with COVID-19 by types of comorbidity: The midpoint of each line illustrates the prevalence; the horizontal line indicates the confidence interval, and the diamond shows the pooled prevalence.

**Fig 5:** Forest plot for prevalence of perioperative complication among surgical patients with COVID-19: The midpoint of each line illustrates the prevalence; the horizontal line indicates the confidence interval, and the diamond shows the pooled prevalence

**Fig 6:** Forest plot for sub-group analysis of prevalence of perioperative complication among surgical patients with COVID-19 by types of complication: The midpoint of each line illustrates the prevalence; the horizontal line indicates the confidence interval, and the diamond shows the pooled prevalence

**Fig 7:** Forest plot for rates postoperative ICU admission among surgical patients with COVID-19: The midpoint of each line illustrates the prevalence; the horizontal line indicates the confidence interval, and the diamond shows the pooled prevalence

**Fig 8:** Forest plot for length of hospital stay among surgical patients with COVID-19: The midpoint of each line illustrates the prevalence; the horizontal line indicates the confidence interval, and the diamond shows the pooled prevalence

**Fig 8:** Forest plot for prevalence of clinical presentation among surgical patients with COVID-19: The midpoint of each line illustrates the prevalence; the horizontal line indicates the confidence interval, and the diamond shows the pooled prevalence

**Fig 9:** Forest plot for subgroup analysis of prevalence of clinical presentation among surgical patients with COVID-19 by types of presentations: The midpoint of each line illustrates the prevalence; the horizontal line indicates the confidence interval, and the diamond shows the pooled prevalence

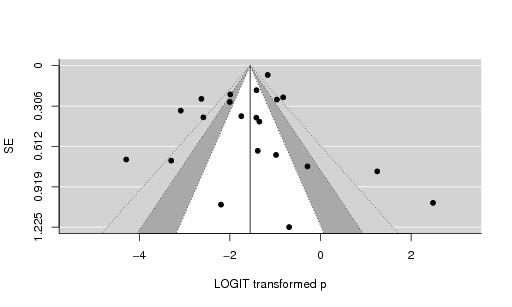

Supplement: Supplementary file 2 — Additional file 2: Supplemental Figure 1. Forest plot for the global prevalence of perioperative mortality among surgical patients with COVID-19 by types of surgery: The midpoint of each line illustrates the prevalence; the horizontal line indicates the confidence interval, and the diamond shows the pooled prevalence. [file 13037_2020_262_MOESM2_ESM.zip › suplemental Fig 1.docx]
