# Supplementary material for: Postoperative mortality among surgical patients with COVID-19: a systematic review and meta-analysis
Source: Patient Saf Surg. 2020 Oct 12;14:37. doi: 10.1186/s13037-020-00262-6 (PMC7549731; doi:10.1186/s13037-020-00262-6)
Supplement: Supplementary file 2 — Additional file 2: Supplemental Figure 1. Forest plot for the global prevalence of perioperative mortality among surgical patients with COVID-19 by types of surgery: The midpoint of each line illustrates the prevalence; the horizontal line indicates the confidence interval, and the diamond shows the pooled prevalence. [file 13037_2020_262_MOESM2_ESM.zip › Supplemental Fig 1.docx]

**Supplemental Fig 1:** Forest plot for the global prevalence of perioperative mortality among surgical patients with COVID-19 by types of surgery: The midpoint of each line illustrates the prevalence; the horizontal line indicates the confidence interval, and the diamond shows the pooled prevalence.
